# Investigation into Effects of Coating on Stress Corrosion of Cable Bolts in Deep Underground Environments

**DOI:** 10.3390/ma17143563

**Published:** 2024-07-18

**Authors:** Saisai Wu, Wanyi Zhang, Jianhang Chen, Krzysztof Skrzypkowski, Krzysztof Zagórski, Anna Zagórska

**Affiliations:** 1 Shanxi Key Laboratory of Geotechnical and Underground Space Engineering, School of Resources Engineering, Xi’an University of Architecture & Technology, Xi’an 710055, China; saisai.wu@xauat.edu.cn (S.W.);; 2 School of Energy and Mining Engineering, China University of Mining and Technology, Beijing 100083, China; 3 Faculty of Civil Engineering and Resource Management, AGH University of Krakow, Mickiewicza 30 Av., 30-059 Kraków, Poland; 4 Faculty of Mechanical Engineering and Robotics, AGH University of Krakow, Mickiewicza 30 Av., 30-059 Kraków, Poland; 5 Anna Zagórska-Research Centre in Kraków, Institute of Geological Sciences, Polish Academy of Science, Senacka 1, 31-002 Kraków, Poland; a.zagorska@ingpan.krakow.pl

**Keywords:** underground engineering, underground soil corrosion, soil corrosivity, stress corrosion, steel strands, cable bolts, coatings, epoxy resin, copper–nickel polymer coating, aluminum-doped asphalt

## Abstract

Due to the intricate and volatile nature of the service environment surrounding prestressing anchoring materials, stress corrosion poses a significant challenge to the sustained stability of underground reinforcement systems. Consequently, it is imperative to identify effective countermeasures against stress corrosion failure in cable bolts within deep underground environments, thereby ensuring the safety of deep resource extraction processes. In this study, the influence of various coatings on the stress corrosion resistance of cable bolts was meticulously examined and evaluated using specifically designed stress-corrosion-testing systems. The specimens were subjected to loading using four-point bending frames and exposed to simulated underground corrosive environments. A detailed analysis and comparison of the failure patterns and mechanisms of specimens coated with different materials were conducted through the meticulous observation of fractographic features. The results revealed stark differences in the stress corrosion behavior of coated and uncoated bolts. Notably, epoxy coatings and chlorinated rubber coatings exhibited superior anti-corrosion capabilities. Conversely, galvanized layers demonstrated the weakest effect due to their sacrificial anti-corrosion mechanism. Furthermore, the effectiveness of the coatings was found to be closely linked to the curing agent and additives used. The findings provide valuable insights for the design and selection of coatings that can enhance the durability and reliability of cable bolts in deep underground environments.

## 1. Introduction

As the shallow layers of the earth’s surface continue to deplete their ore body resources, mining operations will inevitably extend deeper into the subterranean realm. The deep underground environment presents a complex array of challenges, including elevated geostress, geothermal conditions, and increased water influx, all of which heighten the risks associated with the service environment of anchoring materials [1,2]. In such hostile environments, engineering structures must endure unique durability and performance demands. Cable bolts, as crucial components of these structures, are instrumental in maintaining their stability and integrity. Nevertheless, the severe conditions encountered in deep underground settings, including intense stress levels, corrosive soil and groundwater, and fluctuating temperature conditions, can lead to the premature failure of cable bolts. Stress corrosion is one of the primary causes of this failure. This process occurs when mechanical stress and corrosive environments interact, leading to the degradation of the bolt material.

The increasing severity of stress corrosion cracking in deep underground conditions, leading to the failure of rock bolts and cable bolts, has garnered significant attention among researchers [3,4,5]. Hassell et al. [6] conducted a comprehensive study on the corrosion effects of groundwater on anchor rods in eight mining areas in Australia. Their analysis encompassed various factors such as temperature, pH, dissolved oxygen level, total dissolved solids (TDS) concentration, and flow rate, and included EDS analysis. The findings revealed that TDS concentration, dissolved oxygen level, pH, and flow rate are critical influencing factors in determining the corrosion rate of anchor rods. Furthermore, the mining process often exposes specific minerals like pyrite, albite, and magnetopyrite to water and oxygen, resulting in the formation of acidic liquid known as acid mine drainage (AMD). The acidic nature of this liquid, with pH levels reaching as low as 2.3 in some underground mines, has a direct impact on the corrosion rate of anchored materials in their operational environments [7,8,9]. Markovic [10] conducted studies on corrosivity by categorizing geotechnical bodies into three distinct classes: slight, moderate, and severe. These classifications were based on the severity of corrosion, which was influenced by various factors such as grain size, organic content, salt content, pH, resistivity, and water level [11]. Azoor et al. [12] further investigated the corrosion rates of sand, chalk, and clay at varying degrees of saturation. Their findings revealed that each soil type exhibited a unique optimal corrosion rate, with the corrosion rate peaking when this optimal value was reached. Previous research has indicated that there exists an optimal water content in the soil that facilitates maximum corrosion rates of metals. This phenomenon is attributed to the interplay of factors such as electrical conductivity and oxygen diffusion [13,14], as well as the interaction between the active zone and oxygen diffusion. Spearing et al. [15] conducted simulation experiments on anchor materials in various corrosive media under stress corrosion cracking conditions. They observed that the anchor structure was subject to movement caused by the surrounding rock in the roadway, as well as the impact of stress concentration and stripping. This underscores the need for further investigation and the development of effective corrosion prevention strategies to ensure the durability and safety of rock bolts and cable bolts in deep underground mining applications.

Studies on the service environment of anchoring materials have demonstrated that corrosion protection methods that effectively isolate corrosive ions from the metal are beneficial [16,17]. Therefore, it is crucial to investigate strategies for isolating corrosive ions from the metal throughout the duration of underground mining, or at least maximizing the period of isolation. Ideally, when high-carbon steel undergoes industrial processing and comes into contact with a strongly oxidizing environment, the pH of the aqueous environment is maintained at a weakly alkaline state due to the equilibrium established between iron hydroxide and the oxidation process. This equilibrium results in the gradual formation of an oxide film on the metal surface, providing inherent protection through a spontaneously occurring process [18]. However, in the natural corrosive environment of the atmosphere, the corrosion products formed are typically rough and porous, lacking the properties necessary to effectively isolate the steel from the surrounding environment. Consequently, these corrosion products fail to provide protective and self-sustaining qualities. Notably, when corrosion ceases, the oxide film begins to age, dissolve, shrink, or even deteriorate. This deterioration process is accompanied by the separation of anodic and cathodic reaction products, such as iron hydroxide (Fe^2+^ and OH^−^). The anodic portion of iron (II) is oxidized by the air due to a reduction in OH- ions, leading to localized acidification. This process results in crevice corrosion and a continuous decrease in the strength of the metal [19].

The development of novel corrosion protection coatings and inhibitors has garnered significant attention among researchers. Coatings have emerged as a prevalent method to enhance the corrosion resistance of cable bolts. These coatings, constructed from diverse materials like polymers, metals, or ceramics, are applied to the bolt’s surface, creating a protective barrier against corrosive agents. The application process ensures that the coating firmly grips the metal material, effectively isolating it from contact with erosive elements [20,21]. The coating itself exhibits excellent adhesion and ductility. While increased ductility may slightly reduce the bond strength with the concrete layer, it simultaneously enhances the strain capacity of the bond. The corrosion inhibitor works by enhancing the anodic and cathodic polarization behavior, thereby increasing the resistance of the metal surface by mitigating the movement of ions towards it [22]. However, the effectiveness of these coatings under stress corrosion conditions remains incompletely understood. While they may offer some level of protection against general corrosion, their ability to withstand the combined effects of stress and corrosion in deep underground environments is still uncertain.

Therefore, there is a pressing need to delve deeper into the effects of coatings on the stress corrosion behavior of cable bolts. This study endeavored to bridge this knowledge gap by conducting a comprehensive evaluation of the performance of coated cable bolts under stress corrosion conditions. We tested various promising coating methods, including epoxy coatings, chlorinated rubber, and galvanization, using four-point bending experiments with anchor cables as specimens. These experiments were conducted under the condition of simulating the real corrosion environment, and the accurate construction of this simulated environment is a major innovation point of the experiment. It can more accurately reflect the performance of the coating in the actual working environment and provide a more reliable basis for the selection and design of the coating. The study not only focused on the basic protective properties of the coating, but also explored the intrinsic protective mechanism of the coating in depth, and comprehensively analyzed the behavior of the coating through macroscopic and microscopic observations. Furthermore, the durability and reliability of coated cable bolts in deep underground environments were thoroughly accessed. The special characteristics of the underground environment, such as high humidity and high pressure, were taken into account, providing an important reference for the application of coatings in underground engineering.

## 2. Materials and Methods

### 2.1. Specimen Preparation

**Figure 1:**
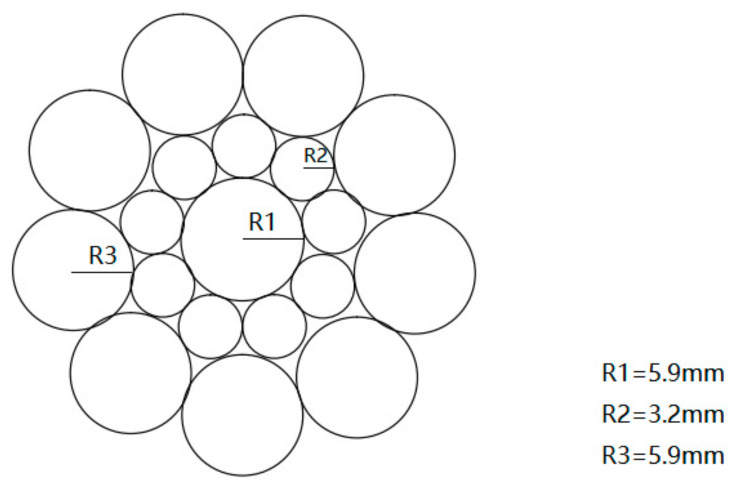
Longitudinal schematic diagram of cable bolt specimen.

For the laboratory tests, a widely utilized cable bolt made by a cold-drawing process commonly employed in deep underground applications was chosen as the experimental specimen. This cable bolt specimen comprised 18 steel strands, including a central straight steel strand. The steel grade of the specimen was designated as SWPR19N, adhering to the Japanese standard JIS G 3536 [23]. The schematic diagram of the anchor cable is depicted in Figure 1. During the design phase of the experimental programs, it was recognized that applying a significant stress–strain to the specimens would be challenging if the full-size cable bolt were utilized. Therefore, the decision was made to utilize the central straight steel strand for the experimental coupon tests. These coupon tests offer notable advantages, including a relatively shorter experimental cycle and more controllable testing conditions, which facilitate the faster acquisition of experimental results. The chemical composition and mechanical properties of the central steel strand used in the experiments are summarized in Table 1 and Table 2.

The application of coatings to cable bolt specimens was carried out using spraying and brushing techniques. The coatings were selected based on their established corrosion resistance properties and compatibility with the bolt material. For the laboratory testing, a range of coating methods were chosen for corrosion resistance evaluation, including hot-dip galvanizing, polyurethane, epoxy coating, Everbrite, Silvershield, chlorinated rubber, and alkyd resin. The specific coatings applied to each specimen and their respective types are outlined in Table 3. Both coated and uncoated bolts were prepared for testing, ensuring that the coating thickness and uniformity adhered to the prescribed standards. Cautions must be taken when preparing the coating, since a tiny “gap” between the coating and bulk specimen could modify the corrosion mechanism. A detailed description of the selected coating methods is provided in the following sections. 

Galvanization stands out as one of the effective anti-corrosion coating methods. It belongs to the category of thick film materials, exhibiting excellent thixotropic properties. After thorough mixing, it can be applied with minimal or no diluent required [24]. The corrosion resistance of galvanization is exceptional, making it a preferred choice as an anti-corrosion material for mines. Epoxy resin, a type of thermosetting resin, has garnered significant attention due to its unique epoxy group [25,26]. This resin features two or more active epoxy groups on its molecular chain. The dense internal molecular structure of epoxy resin and its cured products results in exceptional mechanical properties. When considering coatings for anchor solids, it is crucial to address the potential loss of anchorage force caused by slippage. Epoxy resin excels in this aspect, offering a high degree of adhesion compared to other coatings. Additionally, polar groups like hydroxyl can be utilized as structural adhesives, helping to mitigate the issue of decreased anchorage force. Moreover, epoxy resin boasts stable anti-corrosion properties, a straightforward production process, wear resistance, reduced environmental impact, and lower production costs.

Alkyd resins are oil-modified polyester epoxy resins formulated using polyol and phthalic anhydride [27,28]. Once cured into a film, these resins exhibit a high gloss, toughness, strong adhesion, and impressive abrasion resistance, weather resistance, and insulating properties. However, alkyd resins also have their limitations, including slower film drying, lower hardness, and less-than-ideal water resistance. To enhance their performance, various modification methods are employed, including acrylic resin modification, organosilicon modification, styrene modification, and nanomaterial modification. Chlorinated rubber resin is derived from natural or synthetic rubber through chlorination modification [29]. Its molecular structure is regular, saturated, and exhibits low polarity with no active chemical moieties. This resin is a non-toxic, tasteless, and non-irritating white powder. The introduction of chlorine into the chlorinated rubber resin creates polar C-Cl bonds, chemically inert, that contribute to numerous excellent properties in chlorinated rubber coatings.

Polyurethane, a polymer compound, is continuously broadening its application horizons in the realm of wood furniture coatings and beyond [30]. The latest polyurethane coatings, which have been innovatively developed based on acrylic polyurethane coatings, have found their place in various manufacturing and processing sectors. Everbrite copper–nickel corrosion-resistant alloy stands out as a high-molecular-weight polymer coating, formulated with dipropylene glycol dimethyl ether and ethyl 2-butoxyacetate. Slivershield is an aluminum-doped asphalt, a product of North Corporation, that offers unique properties and applications.

When determining the coating thickness of experimental cable bolts, a number of factors such as protective effect, stress concentration, and adhesion need to be considered. Thicker coatings provide better corrosion protection by isolating the substrate from external corrosive media. An excessive coating thickness may result in stress concentrations between the coating and the substrate or within the coating, which may cause the coating to crack or flake, thus accelerating the corrosion of the cable bolt. The thickness of the coating also affects the adhesion between the coating and the substrate, and an excessively thick coating may result in reduced adhesion. In conclusion, the ideal coating should provide adequate corrosion protection and accommodate the deformation that may occur in the cable bolt during use, as well as have good adhesion and economy.

Before the start of the experiment, a scanning electron microscope was used to observe the microstructure of the coating surface, observing that there were no obvious particles or agglomerations to ensure that the coating was applied uniformly. Then, the coated anchors were subjected to a shear test to measure the force required to shear the coating from the substrate, and the adhesion of the coating was assessed by the magnitude of the shear force. Multiple samples were selected for testing and averaged for final results. It was ensured that the uniformity and adhesion of the coating met the experimental requirements, and the influence of irrelevant factors on the experimental results was exclulded.

### 2.2. Stress Application

**Figure 2:**
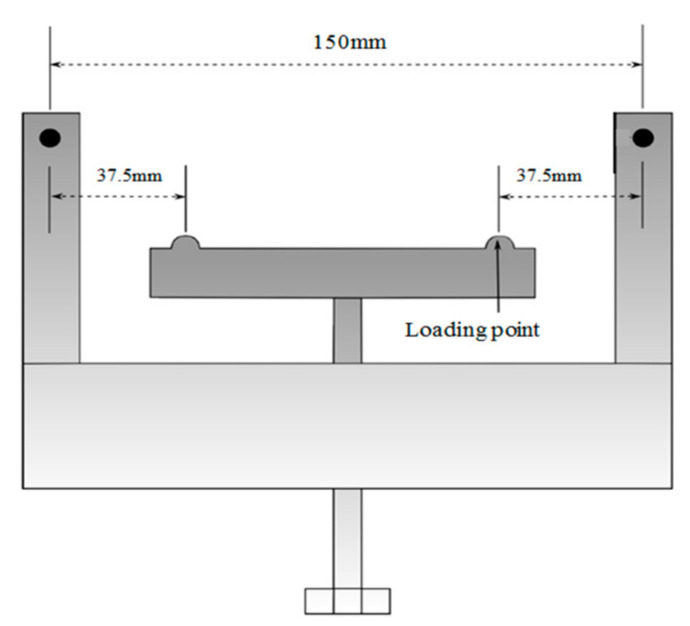
Schematic diagram of the loading apparatus for the four-point pendant experiment.

To replicate the diverse stress conditions encountered in deep underground environments within a controlled laboratory setting, a specialized four-point bend testing apparatus was designed and constructed. This apparatus was capable of applying precisely controlled stress levels, simulating real-world conditions accurately. Figure 2 illustrates the schematic of the loading apparatus for the four-point bending experiment, clearly outlining the geometry of the setup. The outer supports were spaced 150 mm apart, while the distance between the outer and inner supports was set to 37.5 mm. By conducting these four-point loading experiments, bending moments and corresponding tensile stresses were induced in the outer radius of the specimen. The tensile forces generated in this region provided a realistic loading simulation, closely replicating the bending and tensile stresses experienced by anchors in actual underground applications. This approach ensured that the laboratory tests closely mimicked the conditions encountered in deep underground environments, enabling accurate and reliable assessments of material performance. The formula for determining the tensile stresses in the outer fibers of the specimen through analysis is presented in Equation (1). This equation allows for a quantitative understanding of the stress distribution within the specimen, further enhancing the accuracy and reliability of the testing process.

(1) $$\sigma_{4point}=24\;Edy/(3H^{2}-4A^{2})$$

σ = stress in the outer material; E = Young’s modulus of the material; d = distance from the neutral to the outer fiber; y = maximum deflection; H = distance between the outer supports; A = distance between the inner and outer supports.

In the four-point bending experiments, the tensile stresses within the outer fibers of the specimen demonstrated a steady escalation from zero at the outer support columns to a peak value situated between the inner supports. Notably, this peak stress remained consistent throughout the region encompassed by the inner supports. The geometry of the four-point bending frame boasted superior resolution and precision in terms of applied stress, thereby mitigating the likelihood of encountering biaxial stress states that may arise due to factors such as crevice corrosion or loading point contact. Based on the lateral deflection necessary to induce damage in the fractured specimen under compressive loading, the estimated stresses closely approximated 90–95% of the ultimate strength. Additionally, the calculations revealed a minor permanent deformation in the central steel strand at a deflection of 12 mm, indicating that the material had marginally surpassed its yield strength. It should be mentioned that, to avoid the effects of scatter in the tests results, three specimens were conducted for each testing condition. 

### 2.3. Corrosion Environment Simulation

A corrosion chamber was established to replicate the underground environment, encompassing precisely controlled temperature and humidity conditions. Corrosive solutions, mimicking the chemical composition of groundwater or soil typically encountered in mines, were meticulously prepared and introduced into the chamber. It is noteworthy that accurately simulating the anchoring material’s service environment using laboratory means can be challenging, especially when adjusting the proportion of the corrosion solution. The actual service environment of the erosion medium is dynamic and constantly evolving, and cable bolts may have numerous points of contact, including potential surface interactions. The unambiguous objective of conducting simulations in the laboratory is to replicate stress corrosion cracking, ultimately leading to the failure of anchor cables. To achieve this, the preparation of the solution was meticulously designed to enhance its corrosiveness without introducing any biasing effects on the experimental outcomes. This approach successfully accelerated the experimental process while maintaining its controllability and reproducibility. After rigorous research and comparison, a synthetic solution was chosen for this experiment, allowing for the expedited simulation of stress corrosion cracking while ensuring the reliability and reproducibility of the solution medium [31]. The chemical synthesis formula of this solution is outlined in Table 4.

The solutions utilized in this experiment exhibit heightened corrosivity without exhibiting pronounced current characteristics. Given the crucial role of sulfur ions in underground corrosive environments, it has been established that the concentration of H_2_S is a critical factor in the stress corrosion cracking (SCC) process, aligning closely with the solution ratios employed in this study. The hydrogen sulfide required for the experiment was generated via a chemical reaction between sodium sulfide and acetic acid, resulting in a concentration of 0.65 g/L within the solution. Notably, the solubility of H_2_S in a saline solution at 25 °C was approximately 0.084 mol/L, significantly surpassing 0.019 mol/L, indicating its complete solubility within the solution. This solution proved its worth as a reliable simulation of the underground service environment for anchoring materials, effectively inducing stress corrosion cracking [31,32]. Furthermore, the experimental conditions were maintained at a consistent temperature of approximately 25 °C, the pH value was always maintained at around 2.4, and the relative humidity level fluctuated between 80% and 85%.

### 2.4. Experimental Methods

**Figure 3:**
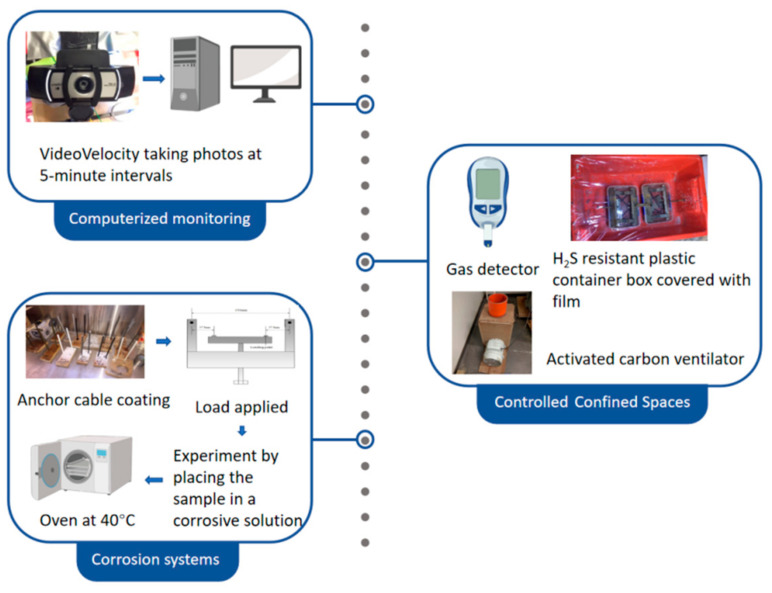
Experimental system diagram.

The experiment can be succinctly categorized into three key components: corrosion systems, computerized monitoring, and controlled confined spaces. The experimental system diagram is clearly depicted in Figure 3. Prior to initiating the experiment, all anchor cables were precisely weighed and organized based on classification labels. The experimental design encompassed five distinct groups, with each group comprising four anchor cables. The coated anchor cables were grouped as outlined in Table 3, and each coated anchor cable was securely placed within the four-point bend testing apparatus. The cable bolts were subjected to a predetermined stress level of 1680 MPa, equivalent to the yield strength of the materials, and this stress level was maintained consistently throughout the entire duration of the experiment. Subsequently, both coated and uncoated cable bolts were submerged in the corrosive solution within the corrosion chamber. The time taken for each specimen to fail was accurately recorded using an overhead video camera, ensuring continuous monitoring throughout the process. Furthermore, photographs were captured at regular intervals of 5 min utilizing the Video Velocity system, enabling the smooth conduct of the experiment even during unmanned evenings and weekends.

Upon completion of the experiments, the bolts were carefully removed from the testing apparatus and underwent a thorough analysis. The specimens that exhibited failure were meticulously cleaned using water and ethanol, thoroughly dried, and then placed in an oven maintained at 40 °C until they were retrieved for further analysis. It is crucial to exercise the utmost precision when applying deflections through the four-point tabs, as minor misalignments in the anchor cables can lead to asymmetrical deflections on opposing sides of the specimen, thereby significantly influencing the experimental outcomes. The coatings were rigorously evaluated for their integrity, adhesion, and corrosion resistance. Non-destructive testing methods, including ultrasonic scanning and scanning electron microscopy, were employed to detect any internal cracks or damage within the bolts. These techniques provide insights into the corrosion mechanisms and the effects of coatings on stress corrosion resistance.

It should be noted that the corrosion solution was prepared within a sealed environment, necessitating the utilization of a gas detector in the laboratory to closely monitor the concentration of hydrogen sulfide gas, given its extreme toxicity to humans. For laboratory analysis, a small aliquot of this solution was dispensed into a beaker for pH assessment, followed by pouring the entire solution uniformly into an H_2_S-resistant plastic container containing the loaded sample. The container was then securely sealed with film to minimize gas leakage. To prioritize safety and mitigate risks to other laboratory personnel, all containers were positioned directly in front of an activated charcoal ventilator, effectively filtering the air to eliminate any excess hydrogen sulfide.

## 3. Experimental Results

**Figure 4:**
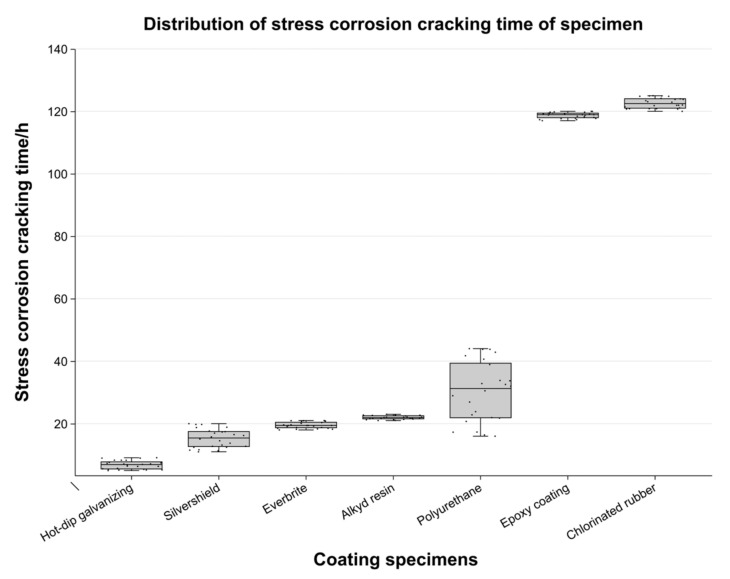
SCC failure time of coated specimens.

Each anchor cable was individually removed and categorized for comprehensive observation records. The primary mechanical tests revealed that the majority of corrosion products were predominantly concentrated in the regions experiencing peak stress–strain. Notably, the coating remained largely intact throughout the four-point hanging experiment, with the exception of the area specifically subjected to deflection. The rupture primarily occurred in the zone where stress–strain was most intense, leading to visible ruptures within the coating area. The exposed metal within the ruptured coating region was in direct contact with the corrosive solution. A statistical analysis of the rupture time distribution was conducted and presented in Figure 4, enabling the calculation of average lifetimes and their sequential ranking. In underground mining projects, variations in ground pressure exert a significant shear force on the anchoring material, thereby necessitating a corresponding level of strain tolerance for the coating layer. While galvanized coatings are effective in mitigating corrosion, their sacrificial nature and cost considerations may render them unsuitable for future underground environments. Furthermore, the limited strain capacity of these coatings can lead to the premature failure of corrosion protection, making them inappropriate for such applications.

Research has demonstrated that epoxy coatings and chlorinated rubber coatings exhibit remarkable corrosion resistance and stability. Chlorinated rubber coatings specifically stand out due to their low water permeability, which effectively minimizes the diffusion of corrosive substances and maintains their properties even at room temperature [33]. However, chlorinated rubber coatings have significant limitations, as they decompose at 130 °C in a dry environment and begin to decompose at 60 °C in a humid environment. Consequently, their practical application is limited to temperatures below 60 °C, greatly restricting their future potential and usability in high-temperature underground environments. Conversely, epoxy coatings possess a high upper limit and promising development prospects. Nevertheless, their lower limit is relatively low, primarily due to the inadequate utilization of their crosslinking properties and the absence of a tight bond with the metal substrate. Based on experimental findings, it is evident that epoxy coatings are more suitable for future research as modified coatings rather than for direct end-use applications.

Polyester resins, as hyper-branched dendritic polymers, possess remarkable properties. However, in this specific experiment, their performance was not particularly outstanding, likely due to a mismatch between their inherent characteristics and the experimental conditions or solution utilized. Conversely, polyurethane elastomers, which occupy a middle ground between epoxy coatings and rubber in terms of performance, offer promising corrosion resistance and robust strain capacity when formulated as PU-based mixtures derived from petroleum. Compared to ordinary alkyd resins, polyurethanes exhibit superior industrial value and are considered a more effective means of corrosion protection. Nevertheless, experimental results for polyurethanes can be highly volatile. This volatility is attributed to the presence of cavities within unmodified polyurethane, allowing the corrosive medium to prematurely come into contact with the anchoring material. The Everbrite and Slivershield coatings failed to demonstrate satisfactory performance in our tests. These two types of coatings are typically employed for household appliances and, as a result, possess limited ductility. Additionally, the Everbrite coatings exhibited the formation of red bubbles, a phenomenon commonly attributed to the presence of hydrogen atoms. The presence of hydrogen atoms is closely associated with stress corrosion cracking, a phenomenon that is highly detrimental to the application of underground anchors. Therefore, given the crucial requirements of high ductility and resistance to stress corrosion cracking in underground environments, these coatings may not be suitable for such applications.

## 4. Material Properties and Microstructural Analysis

### 4.1. Material Properties

**Figure 5:**
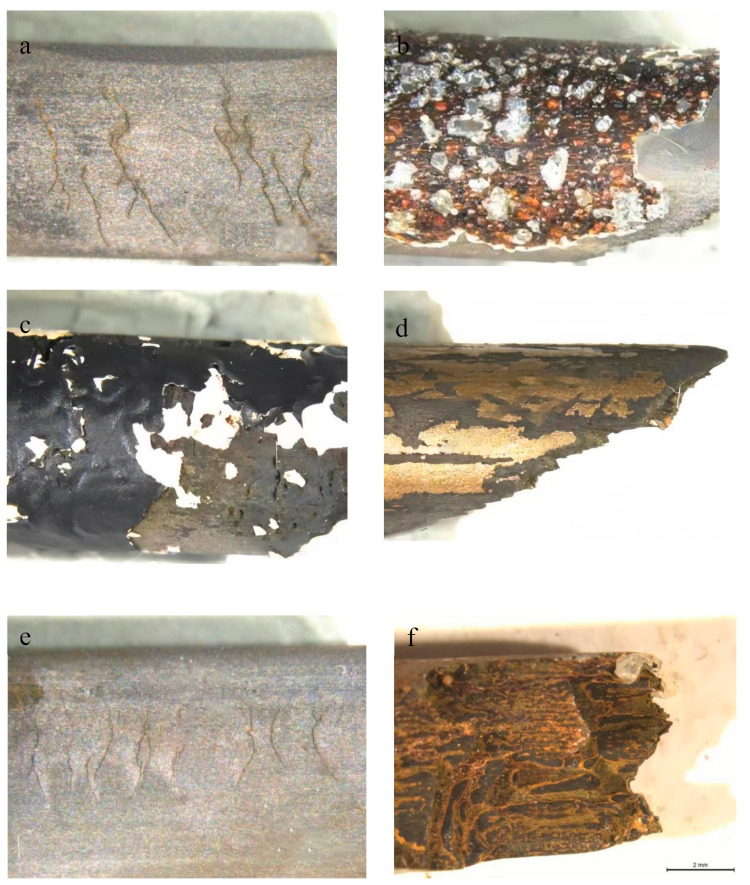
Schematic observation of corrosion results of each coating: (**a**) uncoated; (**b**): Everbrite-coated; (**c**) epoxy-coated; (**d**) Slivershield-coated; (**e**) galvanized-coated; (**f**) center steel fracture surface.

The integrity, adhesion, and corrosion resistance of the coatings were thoroughly evaluated after exposure to the corrosive environment. The collected specimens were meticulously observed and documented using a low-magnification lens, enabling the accurate determination of the corrosion state exhibited by all coatings applied to the specimens. Furthermore, Figure 5 presents a fracture diagram of the anchor cables, captured under a low-magnification lens, offering a visual representation of the fractures observed, providing valuable insights into the coatings’ performance.

In the uncoated anchor cable, distinct subcritical cracks were clearly visible, exhibiting characteristic nucleation and expansion patterns (Figure 5a). Subcritical cracking, a concept derived from fracture mechanics studies, has significantly contributed to advancements in our understanding of stress corrosion cracking [34]. For the Everbrite coatings, it was evident that the coatings exhibited small-scale flaking, revealing the underlying metal interface. Subsequently, red bubbles appeared, imparting a metallic sheen to the coating. These red bubbles may have arisen from a penetration process that interacted with the coating, leading to the accumulation of gases between the coating layer and the metal surface. Notably, no signs of pitting corrosion were observed (Figure 5b), indicating a certain level of corrosion resistance provided by the Everbrite coatings. In the case of the epoxy coating, extensive flaking was observed, revealing large areas of the underlying metal. The white portion represented the primer applied beneath the epoxy coating (Figure 5c). The Slivershield coating exhibited strip-like flaking, exposing the underlying metal with visible black oxide present on the exposed metal surface. However, no apparent cracks or signs of pitting corrosion were observed (Figure 5d). The galvanized coating exhibited notable subcritical cracking, and upon closer inspection, it was discovered that the surface roughness of the metal was significantly smoother compared to the uncoated anchor cable (Figure 5e). This observation suggests that the galvanic coating may have altered the surface characteristics of the metal, potentially influencing its resistance to corrosion and other environmental factors.

From the corrosion observation results of each coating, none of the failed anchor cable specimens exhibited plastic deformation or a reduction in cross-sectional area, indicating a brittle fracture mode of failure. Experimental observations revealed minor deviations in the crack propagation direction. Macroscopically, the fracture path exhibited anisotropy, potentially attributed to the nucleation and expansion of subcritical cracks. Upon examination of the fracture surfaces, it was observed that the primary crack propagated perpendicular to the applied load, with a transverse depth of at least 1 mm. Furthermore, the crack’s evolution progressed from transverse extension to a mixed mode involving bending and longitudinal extension. It is evident that crack initiation occurred at the point of maximum deflection, and subsequently, after a certain extension length, the crack propagated at an angle ranging from approximately 40 to 80 degrees. Prior to ultimate failure, the cracks propagated in a direction closely aligned with the wire’s long axis, exhibiting a characteristic tearing topography surface that has been previously reported by other researchers. The fracture morphology observed in our study aligns with this established behavior, providing further corroboration of brittle fracture occurring in coated anchor cables.

The transformation of the fracture micro-mechanism reveals that cracks propagate perpendicularly to the wire axis, primarily driven by hydrogen embrittlement. Initially, hydrogen diffusion occurs randomly, but due to intense stresses perpendicular to the wire surface, the iron atom bonds at the crack tip undergo increased elastic stretching. Consequently, hydrogen preferentially diffuses towards the crack tip, rather than along the free surface. The accumulation of high concentrations of hydrogen at the crack tip, coupled with the applied axial stress, prompts the crack to propagate directly into the wire, resulting in a tearing morphology fracture micro-mechanism. As the crack penetrates deeper into the material, the available cross-sectional area diminishes, leading to stress concentration at the crack tip. Once the crack attains a critical size, determined by the interplay between the applied stress and the steel’s fracture toughness, rapid brittle failure ensues, occurring along the crack tip as it expands through the highly tensile-oriented microstructure.

### 4.2. Analysis of Microscopic Observations

**Figure 6:**
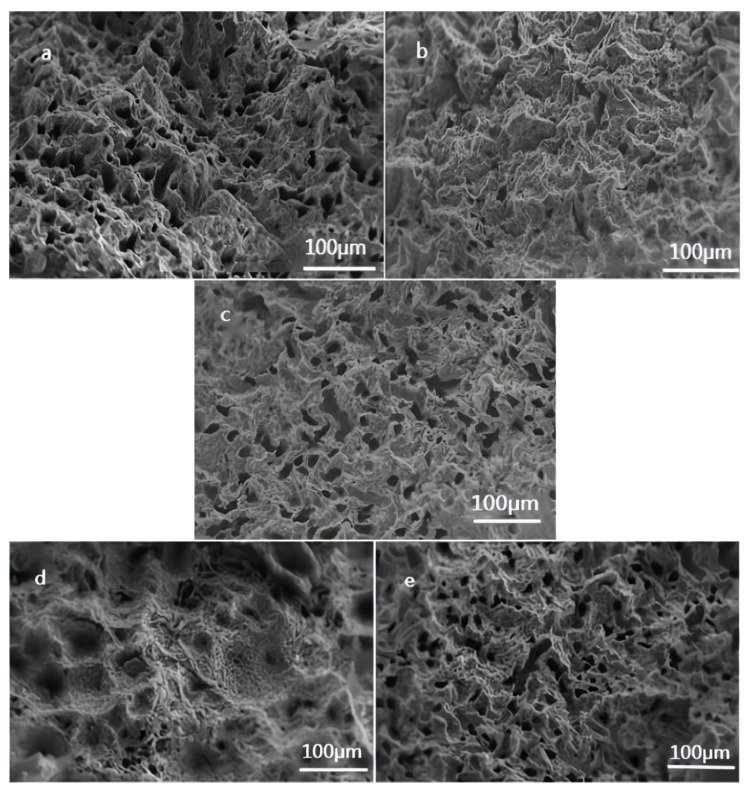
Fracture surface of wires under electron microscope: (**a**) uncoated anchor cable; (**b**): Everbrite-coated; (**c**) epoxy-coated; (**d**) Silvershield-coated; (**e**) galvanized-coated.

The microstructural analysis of the bolts was conducted using scanning electron microscopy (SEM) to meticulously investigate the morphology and distribution of corrosion products and stress corrosion cracks. Upon the examination of the anchor cable’s fracture surface characteristics, a distinct dimple-type fracture pattern emerged. The scanned images revealed numerous tiny micropores scattered across the fracture surface, corresponding to the observed dimples. A comparative analysis of Figure 6 highlighted variations in the number of these dimples. Researchers speculated that hydrogen might have played a role in the nucleation of these holes or the growth of their nuclei. One study found that holes preferentially nucleated along characteristic slip bands, and the presence of hydrogen exacerbated the density of holes in all directions. This observation concurs with our experimental results, where a lower pore count was observed in coated specimens compared to the higher counts in uncoated specimens. This difference could potentially explain the variations in corrosion failure life among the specimens.

Electroplated zinc coatings indeed exhibit remarkable anti-corrosion performance, yet their sacrificial nature and associated costs may render them impractical for future underground applications. A significant concern arises from the coatings’ limited strain capacity, which inevitably leads to the early failure of their anti-corrosion properties. The electroplated zinc layer boasts a tightly bonded surface, where zinc atoms are securely held in place through metal bonds. This arrangement ensures a high degree of regularity and orderliness, resulting in a dense and mostly uniform crystal structure with a smooth surface devoid of voids or concavities. However, the galvanized layer is vulnerable to damage from deflection, resulting in cavities within the bonding surface. These cavities can lead to stress concentration in the anchoring material, ultimately accelerating the corrosion process. The corrosion mechanism involves complex electrochemical reactions, including galvanic coupling and stress corrosion. Notably, achieving a high level of tooth cooperation through mechanical forces in the galvanized layer presents a significant challenge. The comparable number of dimples observed in Figure 6e to the ungalvanized layer further underscores the potential limitations of the galvanized coating in offering significant advantages.

EDS analysis of the anchor cable fractures of the failed coatings showed the presence of a variety of corrosion-related elements and compounds at the anchor cable fractures of the failed coatings. Iron oxides are common corrosion products of iron-based materials in corrosive environments; zinc is used as a sacrificial anode in coatings, and the presence of zinc corrosion products such as zinc oxide and zinc hydroxide are expected. The discovery of elements such as copper (Cu), nickel (Ni), silver (Ag), and titanium dioxide (TiO_2_) suggests that these elements are integral parts of the coating itself. EDS analysis provides information on the specific distribution of these elements in the coating and its fracture. Point, line, or surface analysis provides insight into the relative content and distribution patterns of these elements in different areas. These findings provide important clues about the mechanisms and causes of coating failure and provide a basis for subsequent coating improvements or new material selection.

Various aggressive ions, particularly S, possess a profound ability to inflict damage on stress concentration zones and film rupture zones due to the environmental conditions encountered by the anchoring material. The adsorption of sulfur can significantly hinder passivation processes by accelerating the dissolution of Fe at sensitized grain boundaries or serving as a catalyst that facilitates the ingress of hydrogen into the metal matrix ahead of the crack tip, potentially leading to embrittlement. Reports have documented the theory of grain boundary strain-induced fracture preceding the crack tip in the metal phase, which can significantly expedite the rate of crack propagation [35]. The presence of multidirectional brittle microcracks at the grain boundaries of the metal body serves as evidence of hydrogen-induced fracture. It is noteworthy that even the most robust metal bodies possess inherent defects that cannot be artificially eliminated. Consequently, the selection of corrosion resistance measures must be comprehensive, encompassing multiple capabilities to yield optimal results. This approach aims to mitigate the deleterious effects of aggressive ions and environmental factors on the anchoring material, thereby enhancing its durability and overall performance. 

In corrosive soils, important factors controlling the corrosion process include the interaction between electrical conductivity and oxygen diffusion, and the active zone and oxygen diffusion. The electrical conductivity of the soil is one of the key factors affecting the rate of corrosion. Soil electrical conductivity is affected by a variety of factors such as soil salinity, soil composition, temperature, and water content. Increased soil salinity, fine clayey soils, high water content, and elevated temperatures all increase the electrical conductivity of the soil. For coatings, highly conductive soils may accelerate the electrochemical corrosion process under the coating, so coatings need to have good insulation properties to prevent electrolytes from penetrating the substrate surface. Oxygen is an important participant in the corrosion process, especially in the cathodic reaction, the oxygen content of the soil directly affects the corrosion rate. The corrosion protection performance of a coating is related to its ability to prevent oxygen diffusion. A good quality anti-corrosion coating should be able to form a dense barrier to prevent direct contact between oxygen and the substrate. At the interface between the coating and the soil, active zones may be formed, where the coating is damaged or weak, and these zones are more susceptible to corrosion. Oxygen diffusing into these active zones can react with the metal substrate and accelerate the corrosion process. Therefore, coatings need to have excellent uniformity and integrity to minimize the formation of active zones. In summary, important factors controlling the corrosion process in corrosive soils include the electrical conductivity of the soil, oxygen diffusion, and the interaction between active zones and oxygen diffusion. These factors need to be fully considered when selecting and designing anti-corrosion coatings to ensure that the coatings provide good corrosion protection.

The current study of coating corrosion resistance in this experiment is only at the macroscopic scale, and in the future it is expected to introduce gravity and electrochemical assessment methods to provide further information about corrosion rates.

## 5. Conclusions

In this study, reliable and reproducible data were successfully obtained regarding the impact of coatings on stress corrosion in cable bolts within deep underground environments. This was achieved through meticulous specimen preparation, realistic test condition simulation, and the application of rigorous testing methodologies. Notably, epoxy and chlorinated rubber coatings emerged as standout performers in terms of corrosion protection and stability. The epoxy coating, enhanced by titanium dioxide modification, exhibits remarkable molecular density and cohesion, ensuring robust performance. The chlorinated rubber coating leverages its superior chemical structure to deliver exceptional results. Both coatings demonstrated superior resistance to hydrogen atoms and other corrosive agents, highlighting the significant potential for further developing these coating types. While galvanized layers may not offer the same level of corrosion resistance as some other coatings, their sacrificial mechanism renders them a viable option for emergency anti-corrosion measures. 

The uncoated metal surface exhibited a pronounced formation of subcritical cracks, which displayed a significant tendency to expand and converge extensively. Conversely, the coated anchorage material remained devoid of such cracks. The fractured specimens exhibited distinct tearing patterns, providing valuable insights into the failure mechanisms. The study revealed that the effectiveness of coatings is significantly influenced by both the curing agent and additives used. Single coating materials possess inherent limitations, emphasizing the need to explore alternative solutions. Composite coatings have emerged as a promising approach to address corrosion-induced rupture in deep anchoring materials, offering enhanced performance and durability. The obtained results provide valuable guidance for developing more effective strategies to mitigate the risk of premature bolt failure in deep underground application, which, in turn, contributes to improving the overall stability and safety of underground structures.

## Figures and Tables

**Table 1:** Chemical composition of the specimen (wt.%) (tolerance range ± 1%).

C	Si	Mn	P	S	Mo	Ni	Al	Cr	Cu	Ti	V	Fe
					<					<	<	
0.85	0.31	0.66	0.013	0.009	0.01	0.02	0.02	0.11	0.01	0.01	0.01	Balance

**Table 2:** Mechanical properties of the specimen (tolerance range ± 5%).

Maximum Load	Elongation	Surface Striction (%)	Ultimate Tensile Strength (MPa)	Average Hardness (HV10)
44.37 kN	4.8%	28%	1820	502

**Table 3:** Coatings applied to the specimen and their types.

No.	Coating	Thickness	Type
1	Everbrite	30 μm	High molecular weight polymer coatings containing dipropylene glycol dimethyl ether and ethyl 2-butoxyacetate
2	Epoxy coatings	125 μm	Epoxy coating with titanium dioxide
3	Silvershield	120 μm	Aluminum-doped bitumen
4	Hot-dip galvanizing	70 μm	Galvanized
5	Chlorinated rubber	75 μm	Chemically inert due to polar C-Cl bonds
6	Polyurethane	200 μm	Polyurethane polymer compounds
7	Alkyd resin	45 μm	Oil-modified polyester resins made by condensation polymerization of polyols, phthalic anhydride, and fatty acids or oils (glycerol tri-fatty acid esters).

**Table 4:** Chemical composition of the synthesized solution.

Solute	Molarity/(mol)	Concentration (g/L)
NaCl	0.008	0.45
Na_2_S	0.019	1.5
CaSO_4_	0.007	0.9
CH_3_COOH	0.42	25
pH	2.4	
